# Solid-State Synthesis of Cobalt/NCS Electrocatalyst for Oxygen Reduction Reaction in Dual Chamber Microbial Fuel Cells

**DOI:** 10.3390/nano12244369

**Published:** 2022-12-07

**Authors:** Shaik Ashmath, Hyuk-Jun Kwon, Shaik Gouse Peera, Tae Gwan Lee

**Affiliations:** Department of Environmental Science, Keimyung University, Daegu 42601, Republic of Korea

**Keywords:** oxygen reduction reaction, solid-state synthesis, Co/NCS, nitrogen and sulfur co-doped carbon, microbial fuel cells

## Abstract

Due to the high cost of presently utilized Pt/C catalysts, a quick and sustainable synthesis of electrocatalysts made of cost-effective and earth-abundant metals is urgently needed. In this work, we demonstrated a mechanochemically synthesized cobalt nanoparticles supported on N and S doped carbons derived from a solid-state-reaction between zinc acetate and 2-amino thiazole as metal, organic ligand in presence of cobalt (Co) metal ions Zn_x_Co_x_(C_3_H_4_N_2_S). Pyrolysis of the Zn_x_Co_x_(C_3_H_4_N_2_S) produced, Co/NSC catalyst in which Co nanoparticles are evenly distributed on the nitrogen and sulfur doped carbon support. The Co/NSC catalyst have been characterized with various physical and electrochemical characterization techniques. The Co content in the Zn_x_Co_x_(C_3_H_4_N_2_S) is carefully adjusted by varying the Co content and the optimized Co/NSC-3 catalyst is subjected to the oxygen reduction reaction in 0.1 M HClO_4_ electrolyte. The optimized Co/NSC-3 catalyst reveals acceptable ORR activity with the half-wave potential of ~0.63 V vs. RHE in acidic electrolytes. In addition, the Co/NSC-3 catalyst showed excellent stability with no loss in the ORR activity after 10,000 potential cycles. When applied as cathode catalysts in dual chamber microbial fuel cells, the Co/NCS catalyst delivered satisfactory volumetric power density in comparison with Pt/C.

## 1. Introduction

Microbial fuel cells (MFC) is growing as a promising technology for simultaneous wastewater treatment and bioelectricity generation [1]. The organic matter in the wastewater is converted into H^+^ and electrons by the microorganisms found in the anode. Protons migrate from the anode to the cathode via the proton exchange membrane, while electrons move through the external circuit, which creates the electrical current. The MFC efficiency depends on the type, content and growth characteristics of microorganisms, i.e., bacteria. However, keeping in mind, the real situation, such as large-scale wastewater treatment plants, it is almost impossible to control the type and content of bacteria’s, due to the fact that, the wastewater comes from different sources (ex. domestic/industrial/agricultural, etc.) to the treatment facilities. Additionally, therefore, in order to reduce the cost and complexity, in general, the indigenous bacteria present in the wastewater are utilized for the organic matter degradation and simultaneous power production, in practical situations [2,3]. On the cathode, the protons and electrons combine with the electron acceptor, generally O_2_, to produce water, by oxygen reduction reaction (ORR) [4]. MFC technology is expected to become promising in making wastewater treatment more sustainable and economical in the near future [5]. However, there are a number of problems that severely limit the commercial use of MFC, including effective electron transfer from the bacterial surface to anode electrode, formation of effective biofilm, diffusion of H^+^ from anode to cathode, faster kinetics of cathodic ORR, design, and integration of MFCs that suits to the practical wastewater treatment applications [6]. Among these factors, the kinetics of ORR on cathode, is found to be a factor that greatly affects the performance of MFC. This is due to the fact that ORR is a multi-step and sluggish, especially in acidic electrolytes. Due to its sluggish nature, the ORR demands highly active catalyst such as Pt, therefore, Pt nanoparticles supported on carbon (Pt/C) is considered as the best ORR catalyst so far [7]. However, Pt based catalysts are not ideal for practical application, due to their high cost, poor durability, and scarcity. Recently, transition metal/nitrogen doped carbon-based materials have been demonstrated as one of the best alternative catalysts to traditional Pt/C catalyst for ORR [8]. A number of transition metals such as Co, Mn, Fe, Ni and perovskite-based nanocomposites have been considered as most efficient catalysts for ORR [9,10]. It is believed that transition metals in combination with N form M-N-C (M=Fe, Co, Mn, Ni etc, N-C=nitrogen doped carbon) type of coordination bonds in the catalyst as ORR active centers [11]. Among these catalysts the Fe-N-C type catalysts are considered as the most attractive catalysts due to their remarkable ORR activity in acidic and alkaline electrolytes. However, Fe-N-C catalysts have some limitations such as their ability to form radicals (OH^•^) with the H_2_O_2_ (generated from the 2-electron reduction in O_2_). The OH• radicals are known to degrade the nafion membrane and nafion ionomer in the catalyst layer therefore reduces the ionic conductivity and deteriorates the MFC performance [12]. In this regard, the Co based catalysts are promising due to the fact that Co is inactive for Fenton’s reaction [13].

In general, the M-N-C catalyst are synthesized from the mixture of heteroatom containing organic ligands and metal salts, a carbon source mixture which is then pyrolyzed at a definite temperature to produce M-N-C catalysts [14]. A number of M-N-C catalysts have been synthesized through this universal strategy with a moderate to superb ORR activity [15]. It is to be noted that, most of the M-N-C catalysts synthesis processes involves a definite solution chemistry, in which the precursors are dissolved in a variety of solvents such as ethanol, methanol, N,N-dimethylformamide, toluene, acetic acid, dimethylacetamide, etc. [16]. Due to the use of solution synthesis, obviously a lot of solvent waste will be generated during washing and filtering steps after the reaction completion. Unfortunately, most of the laboratories and small-scale industries have no regulations either to treat or recycle this toxic organic waste therefore creating water pollution subsequently threat to the environment [17]. A recent report from the “European Union-Registration, Evaluation, Authorization and Restriction of Chemicals (EU-REACH)” demanding the chemical industries to shift from organic solvents to green solvents or solvent free synthesis processes due to their toxicity on aquatic species [18]. Hence, it is highly desired to develop solvent free synthesis methods to produce active and stable ORR catalysts for MFC cathodes. In this context, solid-state synthesis techniques is emerging as a fantastic tool to synthesize M-N-C catalysts without use of any solvents during the catalyst synthesis process. Due to their distinctive method of quickly stimulating solid phase reactions, mechanochemistry has been developing for millennia and still has a high level of significance [19]. Solid-state synthesis has a number of benefits over traditional solution-based synthesis methods, including scalability, a higher product yield, solvent-free operations, quick reaction times, and simple handling with a potential of mass manufacturing. Therefore, the solid-state synthesis technique has been utilized for the synthesis of various Fe and Co based catalysts [20].

In this work, we synthesized Co/NCS electrocatalysts via a solid-state, solid-state synthesis process by solid-state reaction with Zn, Co as metal atoms, 2-amino thiazole as the organic ligand and carbon, nitrogen and sulphur source. The precursor Zn_x_Co_x_(C_3_H_4_N_2_S) is subjected to pyrolysis at the temperature of 910 °C to acquire Co/NCS catalyst. The obtained Co/NCS catalyst is characterized by various physicochemical and electrochemical characterizations in 0.1 M HClO_4_. The Co content in the catalysts is systematically optimized and the optimized catalyst with the ideal Co content has been investigated for the ORR kinetics, stability in acidic electrolytes and in dual chamber microbial fuel cell (DCMFC) operated with acetate substrate and atmospheric air as oxidant. It is found that Co/NSC-3 catalyst showed a half-wave potential of ~0.63 V and excellent stability against potential cycling test for 10,000 cycles, almost no loss in half-wave potential. In addition, the Co/NCS-3 catalyst also delivered a volumetric power density of 180 mW m^2^ in the DCMFC set up.

## 2. Materials and Methods

### 2.1. Synthesis of Zn_x_Co_x_(C_3_H_4_N_2_S), NCS and Co/NCS Catalyst

The precursor Zn_x_Co_x_(C_3_H_4_N_2_S) is synthesized by a solid-state synthesis strategy with a laboratory mortar and pestle. The Zn((NO_3_)_2_·6H_2_O, 2-aminothiazole and Co(NO_3_)_2_·6H_2_O are taken in a definite mass ratio (1 g:1 g:0.1/0.150/0.20/0.250 mg, respectively) into a mortar and pestle, and are physically grinded for about 30 min. During the grinding process, the precursors turn into liquid paste and is left at room temperature until it dries to obtain Zn_x_Co_1−x_(C_3_H_4_N_2_S) precursor. The silica crucible is then directly placed at the center of the tubular furnace and then subjected to pyrolysis at the temperature of 910 °C with a heating rate of 5 °C/min under continuous N_2_ atmosphere. Once the desired temperature of 910 °C is reached, the temperature is maintained for 2 h, then the furnace is allowed to cool down naturally to room temperature. A black powder obtained after the pyrolysis are named as Co/NCS-1, Co/NCS-2, Co/NCS-3 and Co/NCS-4, corresponding to the mass ratio of Co(NO_3_)_2_ used during the synthesis (0.1/0.150/0.200/0.250 mg). For comparison, a catalyst is also synthesized without Co(NO_3_)_2_, and the precursor is pyrolyzed at 910 °C, for 1 hr and the catalyst obtained is named NCS. The Co/NCS and NCS catalysts are directly used for physicochemical and electrochemical characterizations without any further processing.

### 2.2. Physical and Electrochemical Characterizations

Particle size, graphitic nature of the Co/NCS and NCS catalysts are studied by using powder X-ray diffraction (XRD, Rigaku (D/Max-2500) diffractometer) analysis with Cu-Kα radiation source (λ = 1.5406 Å). The morphology of the Co/NCS and NCS catalysts are studied by scanning electron FESEM (Hitachi SU8220, Chiyoda City, Tokyo) and transmission electron microscope (FETEM, Titan G2 ChemiSTEM Cs Probe (FEI Company, Eindhoven, The Netherlands). The oxidation state of cobalt and identification of different bonding of N and S are examined by X-ray photoelectron spectroscopy (XPS) using a Thermo Scientific K-Alpha X-ray photoelectron spectrometer.

### 2.3. Electrochemical Characterizations of the Co/NCS and NCS Catalysts

Electrochemical studies of the Co/NCS and NCS catalysts are studied in a traditional three electrode system with glassy carbon electrode (effective surface area of 0.1257 cm^2^) as working electrode, graphite rod and saturated calomel electrode as counter and reference electrodes, respectively. The electrochemical studies were performed by using Biologic instruments SP-150e, potentiostat/galvanostat in 0.1 M HClO_4_. For rotating disk studies, catalyst (Co/NSC or Pt/C) ink is made by dispersing 5 mg of the catalyst in 1 mL of ethanol: water mixture (1:3) and the solution is ultrasonicated for 30 min, to which a nafion solution (5 wt.%) of 15 µL is added and further ultrasonicated for 30 min. A 14 µL of the resultant catalyst ink is deposited in the glassy carbon electrode and then allowed it to dry at room temperature (catalyst loading: 560 µg cm^−2^). For comparison, the Pt/C catalyst (10 wt.%) with a catalyst loading of 110 µg cm^−2^ is also deposited on the GCE.

The ORR electrochemical tests were performed by cyclic voltammetry (CV) and linear sweep voltammetry (LSV). Before the electrochemical tests, the electrolyte 0.1 M HClO_4_ is saturated with N_2_/O_2_ gas. The CV and LSV curves were recorded in the potential range of 0–1.2 V vs. RHE with a scan rate of 50 and 10 mV s^−1^, respectively. For Koutechy-Levich (K–L) plots, the LSV curves were recorded at different rotations speeds from 800 to 2400 rpm. The number of electrons transferred in ORR process at different electrode potentials are calculated from the slopes of Koutechy-Levich (K-L) plots, i.e., plots of *j*^−1^ vs. ω^−1/2^ using the following questions.

(1) $$\frac{1}{j}=\frac{1}{j_{k}}+\frac{1}{j_{L}}$$

(2) $$\frac{1}{j}=\frac{1}{j_{k}}+\frac{1}{B\omega^{1/2}}$$

(3) $$B=0.62\;n\;FC_{O_{2}}D_{O_{2}}{}^{2/3}\upsilon^{-1/6}$$

where *j* is measured current density, *j_L_* is diffusion-limiting current density, *j_k_* is kinetic current density, ω is angular velocity (rad s^−1^). *B* is a parameter calculated from Equation (3); “*n*” is number of electrons transfer per O_2_ molecule, *F* is Faraday constant (96,485 C mol^−1^), $C_{O_{2}}$ is the concentration of oxygen in electrolyte $1.2\times 10^{-6}{\;\mathrm{mol}\mathrm{cm}}^{-3}$, $D_{O_{2}}$ is diffusion co-efficient of oxygen in the solution ($1.9\times 10^{-5}{\mathrm{cm}}^{2}s^{-1}$) and υ is kinematic viscosity of 0.1 M aqueous KOH electrolyte (0.01 cm^2^ s^−1^). The stability of the Co/NCS and Pt/C catalysts have been carried out by potential cycling of the working electrode by recording repeated cyclic voltammograms between 0 to 1.0 V vs. RHE for 5000 and 10,000 cycles at the scan rate of 50 mV s^−1^. To assess the degradation of the catalysts, the LSVs were recorded after stability test in O_2_ saturated 0.1 M HClO_4_ electrolyte with a scan rate of 10 mV s^−1^. All the potential was represented on relative hydrogen electrode (RHE) scale for convenience.

### 2.4. Microbial Fuel Cell Operation

The MFC reactor used was a dual chamber glass reactor with a volume capacity of 250 mL each (with a working volume of 200 mL). The carbon brush was used as an anode. The as received carbon brush was washed with ethanol and water to remove any dust and impurities. The two glass chambers are separated by a proton exchange membrane (Nafion 117). The as received membrane was pre-treated in 30 wt.% H_2_O_2_, then in 0.5 M H_2_SO_4_ and finally in deionized (DI) water. Each step is performed for 1 h at 80 °C. The pre-treated membrane is then stored in DI until use. The cathode used was a commercial GDL (geometric area = 2.6 × 5.5 cm). The cathode catalyst Co/NSC and Pt/C in the form of catalyst ink is deposited onto the GDL. The cathode catalyst ink is prepared by dispersing definite of the Co/NSC catalyst in isopropyl alcohol and ultrasonicated for 30 min, to which 30 wt.% of Nafion (5 wt.%) solution is added and further ultrasonicated for 30 min to obtain the final catalyst ink. The catalyst ink is then coated onto the GDL by using paint brush layer by layer until a desired catalyst loading 2 mg cm^−2^ for both Co/NSC catalyst while 0.5 mg cm^−2^ in case of Pt/C catalyst is achieved. The two MFC chambers were coupled with the Nafion 117 membrane, and two chambers are filled with 200 mL of the buffer solution (pH = 7) on both sides. The anode chamber consists of 20 mM sodium acetate. The cathode is purged continuously with atmospheric air with the help of an air pump at a controlled flow rate (~10 mL min^−1^). In this work, we have “utilized bacterial seed sample”, from sewage water, collected from the local drainage as the source of bacteria. A sample of 10 mL sewage drainage water sample is added to the anodes of fresh MFC set up, allowing the bacteria to culture naturally in the anode chamber. Sewage water is a good source of mixed bacterial strains and mimics the most realistic wastewater treatment applications. In addition, as a source of carbon, we add 20 mM sodium acetate every alternative day to the anode chamber and allow the bacteria to grow. Over the course of time, bacteria attaches the carbon brush fibers and grow continuously, forming a biofilm [21,22]. The biofilm formation can be assessed from the fact that the OCV slowly rises and reaches the maximum after a period of time. After reaching a steady and stable OCV, then the polarization curves were recorded. The anode and cathodes are connected to the potentiostat to monitor the open circuit voltage. The polarization curves were recorded by linear sweep voltammetry at a scan rate of 1 mV s^−1^. The current density and power densities were normalized according to the cathode geometric area and volume of the anolyte.

## 3. Results

A simple one step solid-state synthesis technique was adopted to synthesize Co/N-C-S catalyst. Conventionally, M-N-C catalysts are synthesized by the traditional co-ordination reaction between 2-methyl imidazole, as organic ligand with a zinc/cobalt as metal nodes in a methanolic solution, which yields a precipitate over a reaction time and the precipitate needs to be washed and centrifuged [23]. In this study, a simple, quick, one pot synthesis strategy is developed in which the precursors are simply grinded in the mortar and pestle. During the grinding processes, mechanical energy favors the formation of co-ordination bonds to build the metal organic framework (MOF) like structures just as a methanolic solution reaction does [24,25,26,27]. There are a number of researchers who explored solid-state techniques to synthesize MOFs as can be seen from the literatures [28,29,30]. Even though it is well known that MOFs with N-heterocyclic organic ligands are often employed, there are not many investigations on MOFs with more than one heteroatom in the heterocyclic ring. For instance, the most popular ligand for organic compounds containing N is 2-methyl imidazole [31,32]. In addition to altering the electrical distribution, the presence of many heteroatoms in a single organic linker also affords efficient metal coordination sites during the synthesis of MOFs [33]. When creating MOFs, the presence of several heteroatoms in organic linkers helps to improve bond polarization which further helps in boding with metal ions. Electron-rich atoms, such as nitrogen and sulfur, further assist in unique interactions that occur during the solid-state synthesize of MOFs. These interactions include hydrogen bonding and π-π stacking, both of which contribute to the construction of stable, fascinating supramolecular architectures [33]. Thiazoles and thiazolidines, which promote coordination with both soft and hard metals from the periodic table, are especially attractive N, S-containing heterocyclic compounds for the aim of developing MOFs since they provide a wonderful opportunity to synthesis a range of MOFs [34,35].

**Scheme 1:**
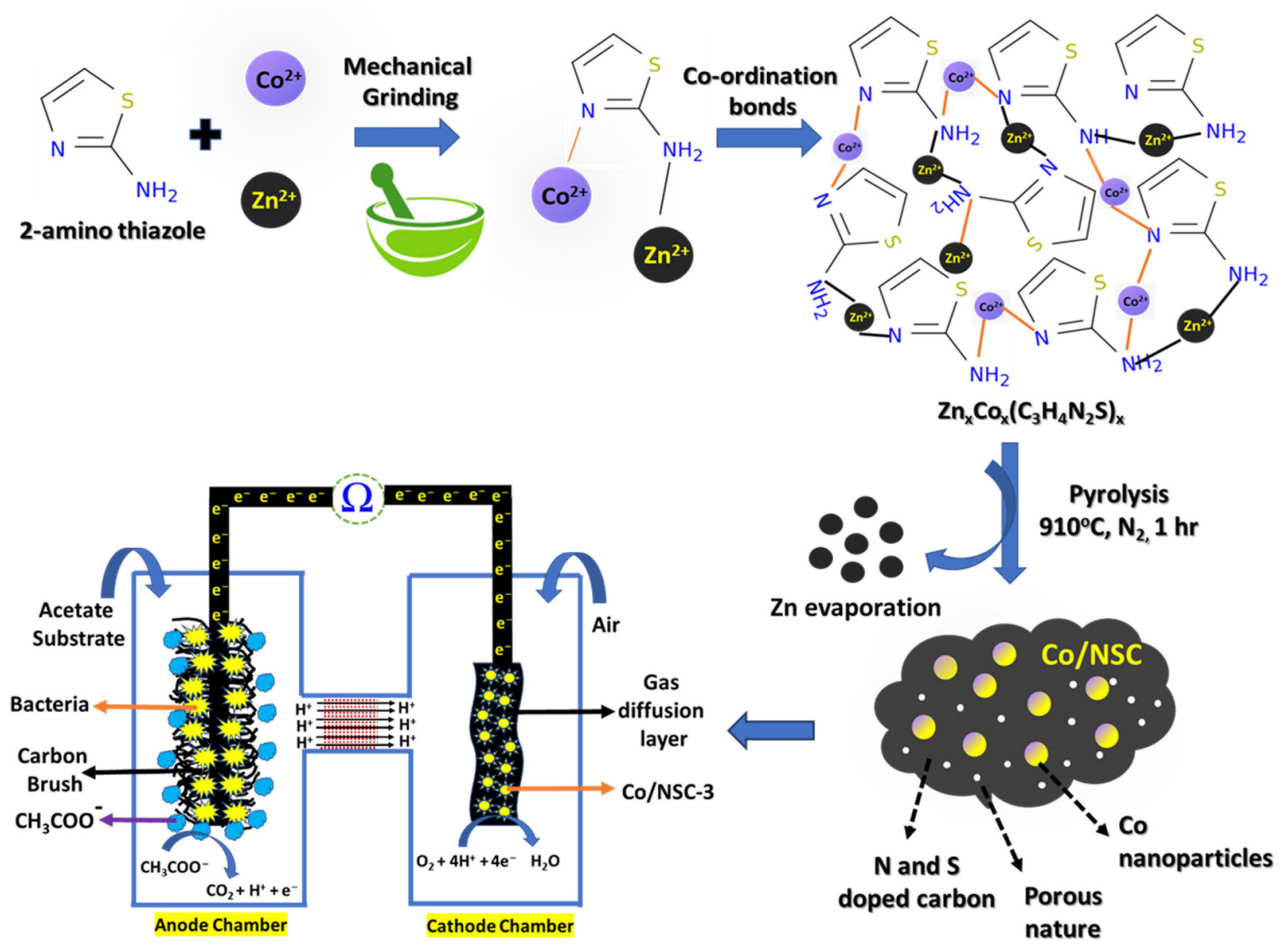
Graphical representation of the Co/NSC catalyst synthesis process and its application in DCMFC.

In this work, we used a solid-state synthesis method to create MOFs employing a thiazole organic ligand, namely 2-aminothiazole, with Zn^2+^ ions and Co^2+^ ions. This study was inspired by the assertions that were made above. In brief, calculated amount of zinc acetate, 2-aminothiazole with varying amount of cobalt nitrate are taken in a mortar and ground for around thirty minutes. This produces a thick pink color paste that, once dried, which results in the formation of possible intermediate precursor, Zn_x_Co_x_(C_3_H_4_N_2_S), analogues to Co-MOF. The unique property of 2-aminothiazole is that it reacts in its neutral state, with the nitrogen atom in the ring acting as the more reactive center. The ring N and amino N, both involve in the resonance to form co-ordination bonds with various electrophiles such as transition metal atoms such as Co^2+^/Zn^2+^ ions to form intermediate Zn_x_Co_x_(C_3_H_4_N_2_S) [36]. During the pyrolysis of intermediate precursor, Zn_x_Co_x_(C_3_H_4_N_2_S) in the presence of a N_2_ atmosphere, the 2-aminothiazole ligand decomposes, graphitizes, and eventually converts into N, S-doped carbon. This occurs simultaneously with the carbo-reduction in the Co^2+^ ions into Co metal and Co-N-C active sites, as well as the loss of Zn to ultimately result in the desired Co/NCS catalyst, in which the cobalt nanoparticles are evenly distributed throughout the N and S-doped carbon support [Scheme 1]. The precursor Zn_x_Co_x_(C_3_H_4_N_2_S) is synthesized by varying composition of Co^2+^ precursors (0.1/0.150/0.20/0.250 mg) and are pyrolyzed at 900 °C, under N_2_ atmosphere to obtain the Co/NCS–1, 2, 3 and 4, respectively. The obtained Co/NCS catalysts are characterized by various physical and electrochemical techniques as explained below.

**Figure 1:**
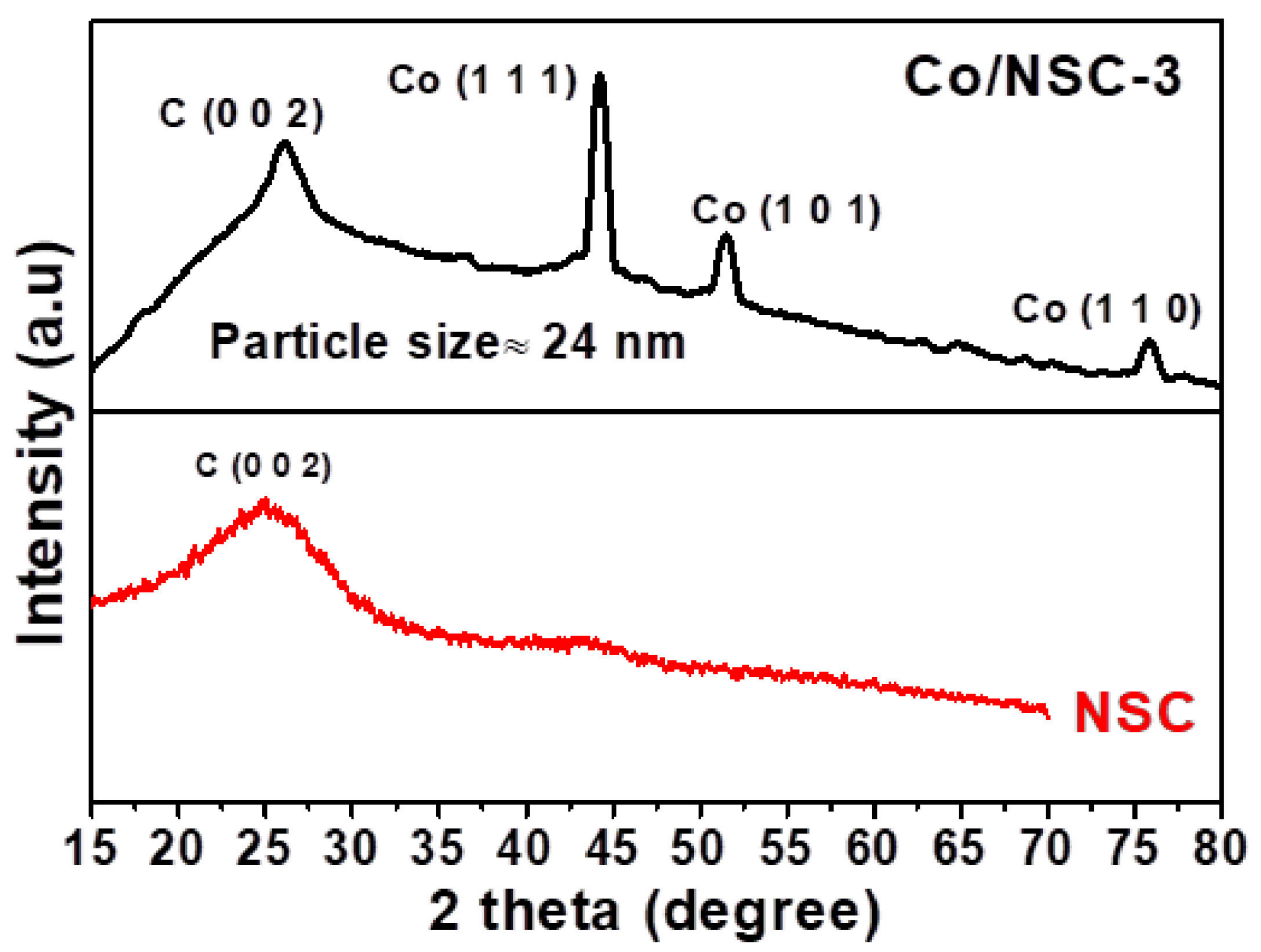
XRD patterns of NSC and Co/NSC-3 catalysts.

**Figure 2:**
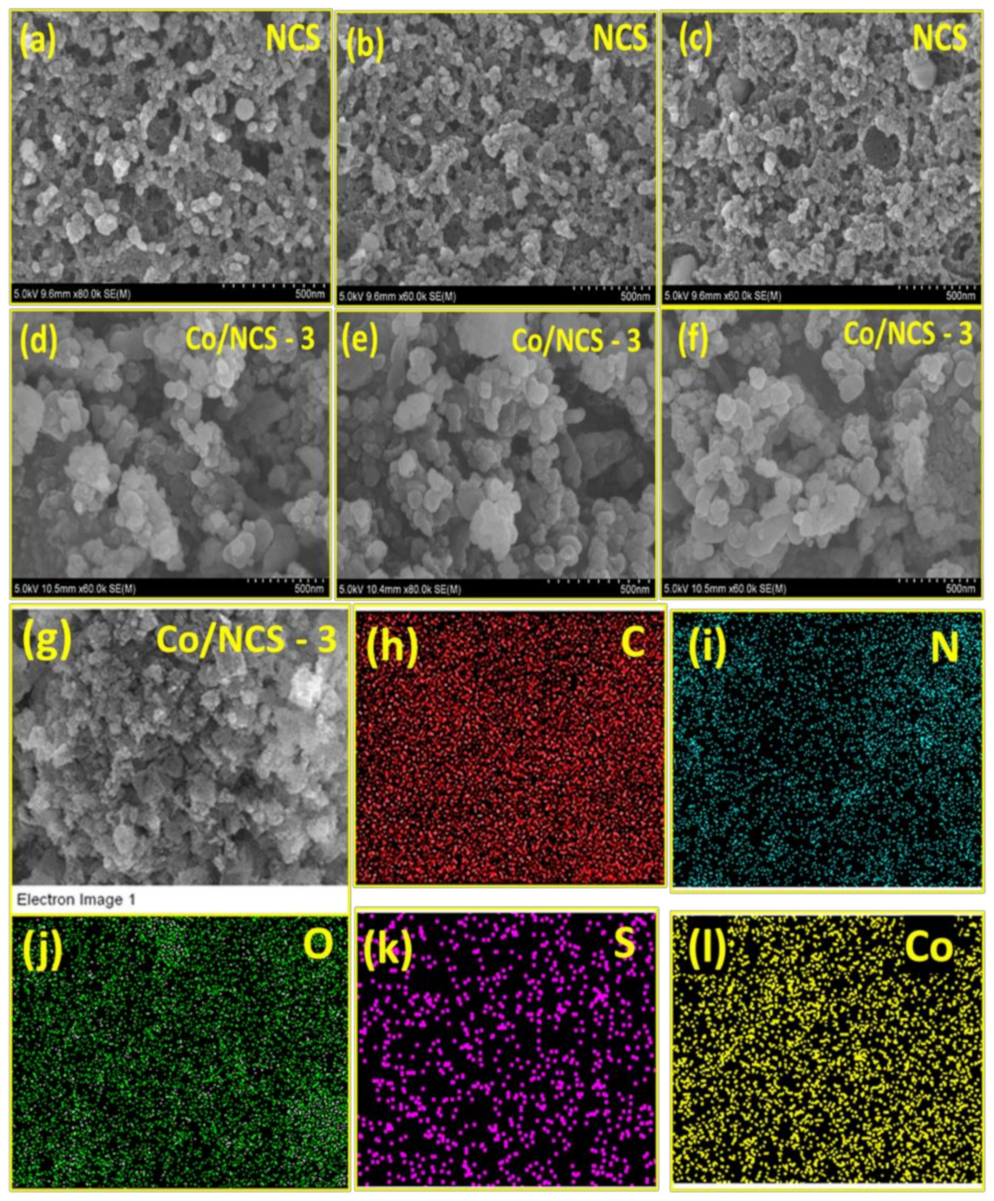
SEM images of NCS (**a**–**c**), Co/NSC-3 (**d**–**f**) and SEM elemental mapping for Co/NSC-3 catalyst (**g**) images area (**h**) mapping for C (**i**) N (**j**) O (**k**) S (**l**) Co atoms.

The XRD analysis was performed to identify the phases and crystal size of Co nanoparticles. Figure 1 shows the diffraction pattern of Co/NSC-3 which show diffraction peaks around ~26 (2θ) values corresponding to the carbon (0 0 2) plane, which results from the decomposition and graphitization of the 2-aminothiazole ligand into carbon. At the same time, the Co-NSC-3 sample showed the obvious peaks at the 2θ values of 44°, 51°, and 75° represent the metallic Co (111), Co (101), and Co (110) phases, respectively [37]. The average crystallite size of Co/NCS-3 was calculated using the Scherrer formula, by taking Co (111) phase, the results came out to be ~ 24 nm, respectively, for Co/NCS-3. Field emission scanning electron microscopy (FE-SEM) and transmission electron microscopy (TEM) techniques are used to analyze the morphological observations of the catalysts. Figure 2 shows the SEM images of the pyrolyzed Zn_x_(SN_2_H_4_C_3_) and Zn_x_Co_x_(C_3_H_4_N_2_S) which results in the formation of NCS and Co/NCS catalysts, respectively. It is observed that NCS catalyst (Figure 2a–c) showed a spherical morphology of the carbon derived from the pyrolysis of Zn_x_(SN_2_H_4_C_3_). In contrast, the Zn_x_Co_x_(C_3_H_4_N_2_S) derived catalyst, i.e., Co/NSC-3 (Figure 2d–f) showed larger particles of carbon, possibly due to presence of cobalt which induces the graphitization of the carbon during the pyrolysis process which results in increased particle size. Figure 2g–l shows the FE-SEM elemental mapping of the Co-NCS-3 catalyst, which confirm the presence of C, N, O, S and Co in the sample, especially presence of N and S indicates the successful doping of N and S into carbon matrix.

**Figure 3:**
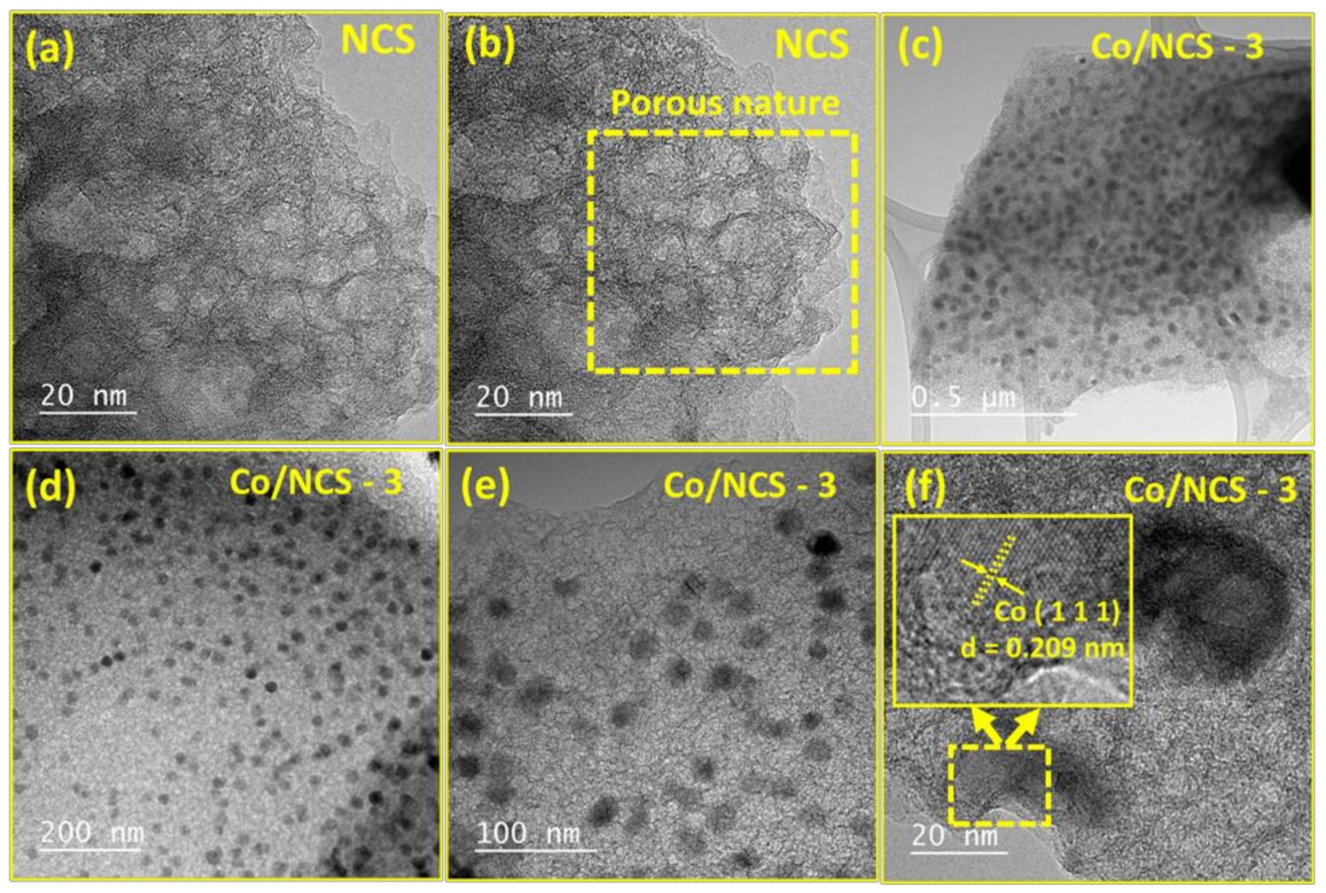
TEM images of NCS (**a**,**b**), Co/NSC-3 (**c**–**f**). The yellow box in figure (**b**) indicates the porous nature of NCS carbon. Inset of (**f**) shows the lattice fringes with the d = 0.209 nm corresponds to Co (1 1 1).

Figure 3 shows the TEM images of the NCS and Co/NCS catalysts. Figure 3a,b shows the TEM images of the NCS catalyst. It is clearly seen that a porous carbon derived from the pyrolysis of Zn_x_Co_x_(C_3_H_4_N_2_S) catalyst, in which the 2-aminothiazole decomposes into carbon network. During the carbonization process, Zn^2+^ ions converts into ZnO (<750 °C), At carbonization temperatures > 750 °C, the ZnO clusters were reduced to metallic Zn through carbothermic reduction (ZnO + C → Zn(l) + CO(g)). Subsequently, further increasing the temperature to 910 °C, the Zn metal clusters (boiling point: 908 8C) were vaporized, along with the inert gases, Zn(l) → Zn(g). The pores in the carbon matrix are left by the evaporated gaseous Zn. As a result, Zn works as a porogen (porous inducing agent) in the carbon matrix [38,39]. The carbon’s porosity makes it easier for gaseous O_2_ to reach the catalytic active site while also facilitating the rapid removal of product water, which lowers the mass transfer limitations during the oxygen reduction reaction. Figure 3c–f shows the TEM images of the Co/NCS-3 catalyst which clearly shows the nanosized metallic Co nanoparticles are evenly distributed on the porous N and S doped carbon support, with the particle size between 8–27 nm, with the average particles are in the range of ~18–20 nm (Figure S1). The Co nanoparticles’ lattice fingers were determined to be Co (1 1 1) planes with an interlayer spacing of 0.209 nm (inset of Figure 3f).

**Figure 4:**
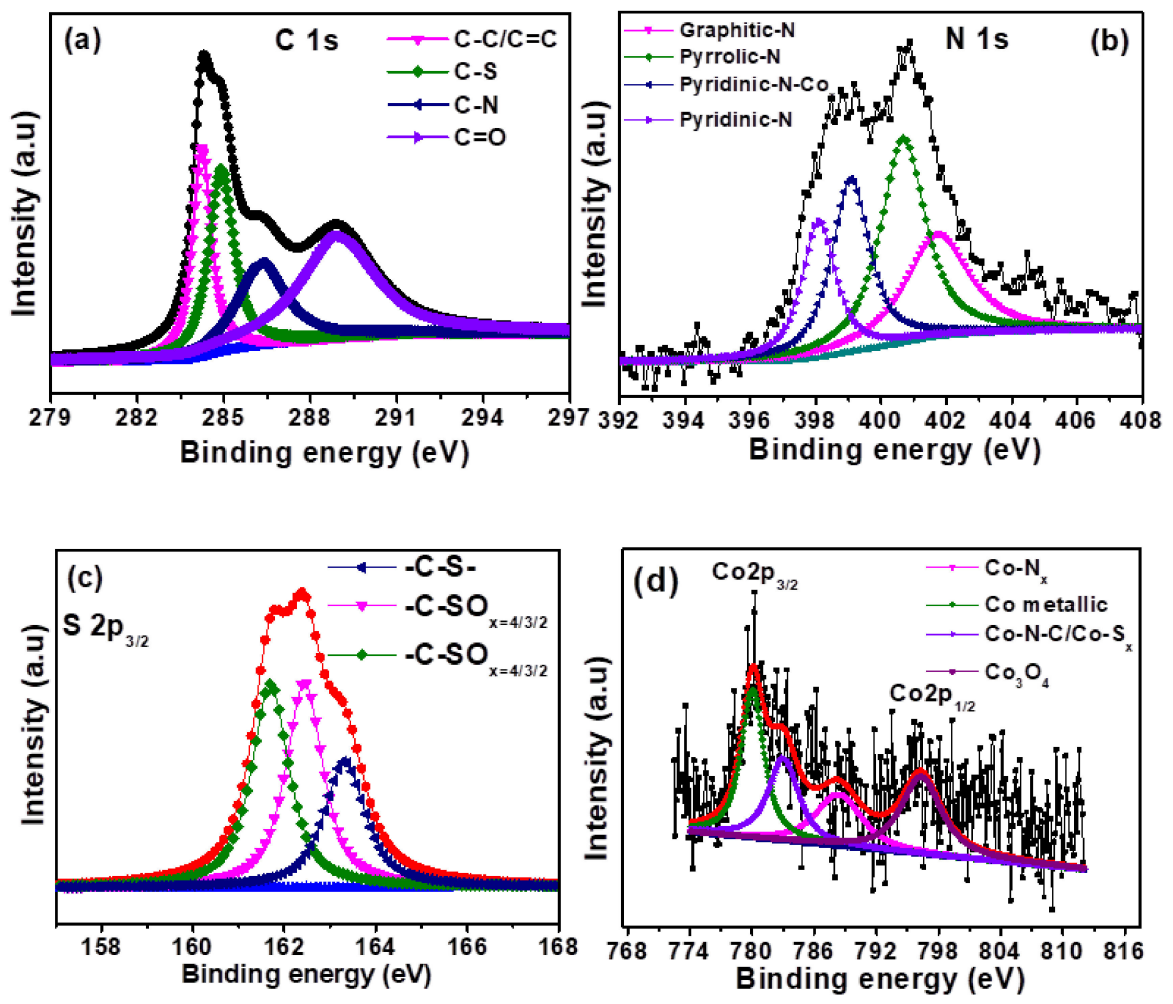
XPS deconvoluted spectra for (**a**) C 1 s (**b**) N 1 s (**c**) S 2p^3/2^ (**d**) Co 2p spectra.

To identify the chemical composition and chemical states of the Co/NCS catalyst, XPS analysis is carried out. The XPS survey spectra (Figure S2a) shows the presence of C, N, S and Co in the catalysts. Further to understand the chemical states, each spectrum is recorded again with a high-resolution scan and deconvoluted. Figure 4a shows the deconvoluted spectra of the C1s which reveals four distinctive peaks at the binding energies of 284.25, 284.9, 286.3, 288.9 eV, corresponding to the bonding of C-C/C=C, C-S, C-N, C=O, respectively [40]. The presence of -C-N- and C-S- bonds strongly suggests that the Co/NSC-3 catalyst possesses nitrogen and sulfur and confirms their successful incorporation into the carbon matrix following the decomposition of 2-aminothiazole. Figure 4b shows the deconvoluted N1s spectra which showed four different deconvoluted peaks at the binding energies of 398.08, 399.08, 400.68 and 401.78 eV corresponding to the N doping in the form of pyridinic-N, pyridinic-N-Cox, pyrrolic-N and graphitic-N, respectively [41]. 

By altering the electroneutrality of the carbon atoms, graphitic-N and pyridinic-N species are known to be the potential active sites for O_2_ adsorption and its reduction to H_2_O [42]. However, there exists a lot of controversy whether the pyridinic, graphitic, or both nitrogens’ are active sites for ORR. Some researchers believed that pyridinic N acts as effective active centers to boost electrocatalytic ORR performance [43,44], but others believed that graphitic N, rather than pyridinic N, contributes significantly to ORR performance enhancement [45,46]. Kim et al. proposed a mutual transition between graphitic N and pyridinic N using a cyclic C-N ring opening method to resolve this problem [47]. Due to simultaneous formation of different N species and lack of precisely controlled experimental techniques on type of N species, makes it a difficult task to in elucidating the unique active site. Therefore, it is believed that the synergistic effect of pyridinic-N and graphitic-N are responsible for ORR [48] Additionally, by contributing their electrons to the carbon network and thereby increasing the electron density around Co nanoparticles, pyridinic-N and graphitic-N species can also improve the interaction between O_2_ and Co nanoparticles in Co/NSC-3. Due to the high electron density around metallic Co catalytic centers, the work function of Co is reduced, which in turn leads to an improvement in the ORR kinetics. In addition to the pyridinic-N, appearance of a distinctive peak ~399.08 eV also suggests that pyridinic-N may also coordinate with Co metallic atoms, therefore, giving rise to a potentially active ORR sites namely M_x_-N-C (Co_x_-N-C) [49]. Numerous theoretical and spectro-analytic studies evidenced that existence of Cox-N-C significantly enhances the ORR by a direct 4 electron transfer [50]. Therefore, presence of pyridinic-N, graphitic-N and Cox-N-C synergistically enhances the ORR activity. Figure 4c shows the deconvoluted S 2p^3/2^ into three different peaks at the binding energies of 161.68, 162.48 and 163.28 eV. The former two peaks corresponds to the existence of -C-SO_x_ (x = 4/3/2) species and the later peak corresponds to the presence of -C-S-C bonds [51]. The evidence of -C-S-C bonds clearly indicates the effective doping of S atoms into the carbon matrix. It is well established that S atoms increases the spin density of the carbon atoms surrounding the S dopant, which boosts the ORR reactions [52]. The C-SO_x_-C bonding further shows that S dopant plays an important role in adsorbing O_2_ atoms during the ORR process. Figure 4d shows the deconvoluted Co2p spectra, which show five peaks at the binding energies of 777.4, 780.1, 782.6 and 796.1 eV, corresponding to metallic Co, Co-N-C/Co-S_x_, CoN_x_, and Co_3_O_4_ peaks, respectively [53]. The existence of Co-N-C and Co-N_x_ species in the Co/NSC catalyst is further confirmed by the occurrence of peaks corresponding to Co-N atoms. Since S atoms are also present in the carbon matrix, it is impossible to rule out the potential of Co species (Co-S_x_) forming bonds with S atoms. There is sufficient evidence to suggest that the addition of S atoms to M-N-C active ites further enhance the ORR kinetics by altering the electronic structure of the of M-N-C sites catalytically active centers. 

**Figure 5:**
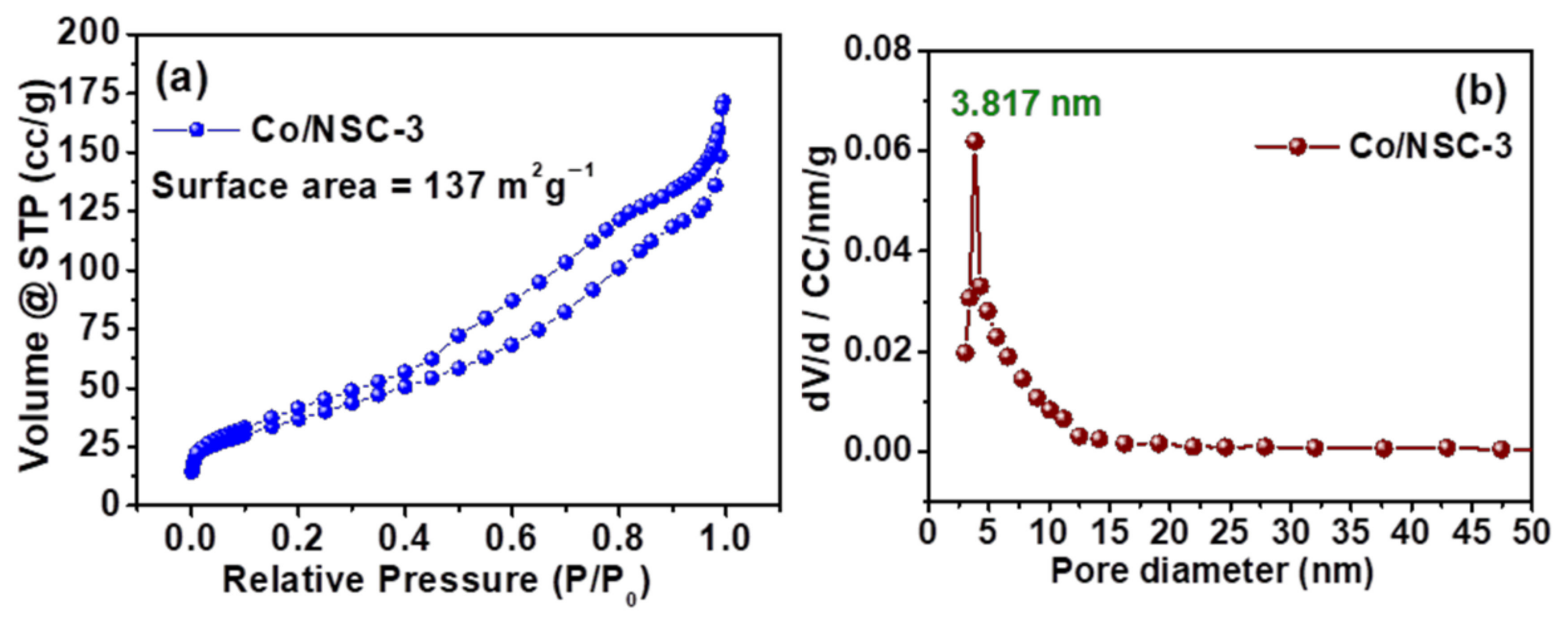
(**a**) N_2_ adsorption/desorption isotherm curves of Co/NSC-3 and (**b**) pore size distribution curves of Co/NSC-3 catalyst.

Figure 5 demonstrates the N_2_ adsorption and desorption isotherms as well as the BJH pore size distribution curves. The existence of mesopores in the catalyst is indicated by the fact that the Co/NCS-3 catalyst displays an isotherm of the type IV kind with a hysteresis loop ranging from a relative pressure of 0.40 to 1.0. The BET surface area that was achieved is 137 m^2^ g^−1^, and the mesopores that are present in the Co/NCS-3 catalyst are mostly between 3 and 15 nm in size, with a maximum number of pores of 3.817 nm. It is well knowledge that the existence of mesopores is largely significant for the increased ORR performance because it helps to strike a balance between the mass transfer of reactants towards the catalytic active sites and the removal of product water from the catalyst layer.

**Figure 6:**
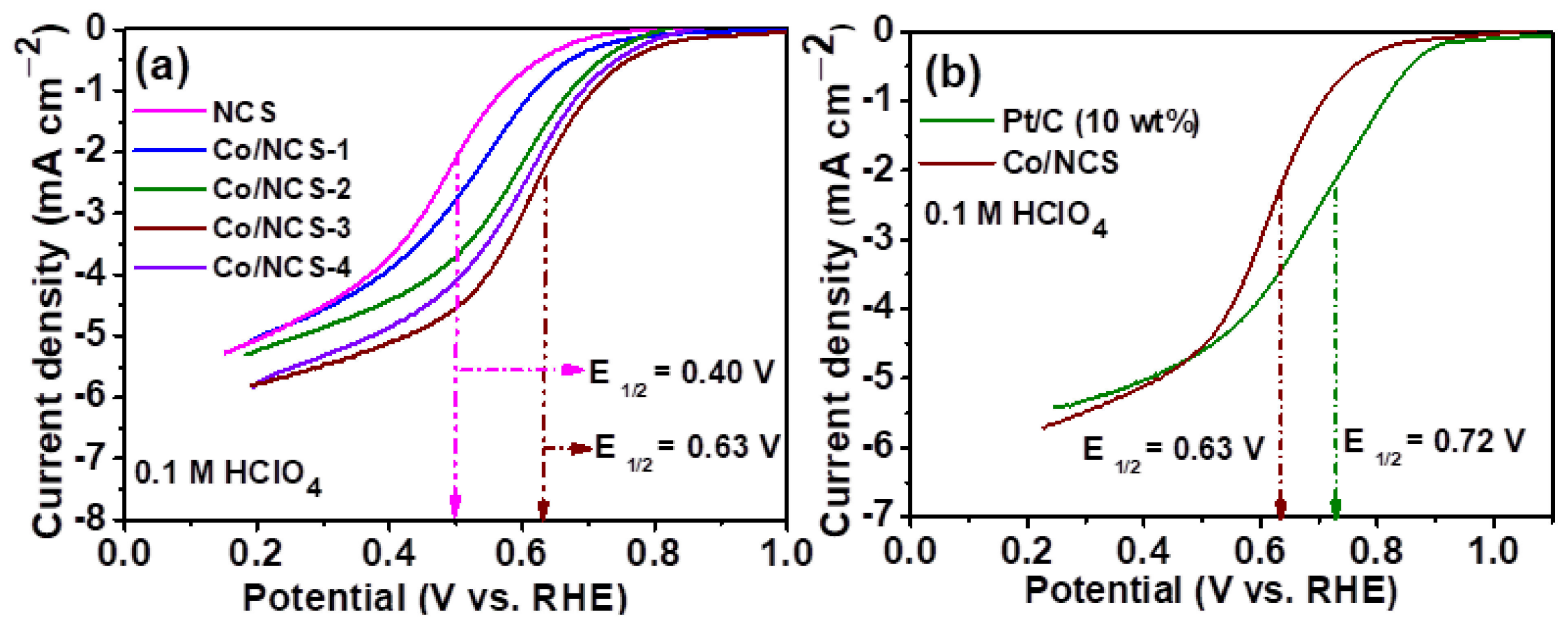
(**a**) Linear Sweep Voltametric (LSV) curves of Co/NSC catalysts with different Co contents (**b**) Comparison of LSV curves of Co@NSC-3 catalyst with commercial Pt/C (10 wt.%). Electrolyte: 0.1 M HClO_4_, scan rate: 10 mV/s.

After ascertaining the morphology and chemical nature of the catalysts, the ORR activity of Co/NCS catalysts have been evaluated for ORR in 0.1 M HClO_4_ electrolytes. Figure 6a shows the ORR curves for Co/NCS catalysts synthesized with different Co contents. The catalyst is also prepared without Co for the ascertaining the role of Co. All the catalysts showed a typical characteristic of an ORR curve, with a well definite kinetic region, mixed kinetic diffusion-controlled region and limiting current regions, indicating that al the catalysts are active for ORR. Half-wave potential (E_1/2_), i.e a potential at which ORR curve is equal to one half of the limiting current, is calculated to assess the superiority of one catalyst over the other. The catalyst synthesized with 2-amino thiazole (NCS), showed the E_1/2_ of 0.40 V vs. RHE. The ORR activity of NCS catalyst is due to the presence of various N active sites in the catalyst such as graphitic, pyridinic and pyrrolic-N species, together with spin density induced kinetics by C-S-C active sites. Interestingly, the ORR activity of the NCS catalyst increases significantly when Co is introduced during the synthesis process. This is clearly evidenced from the increased ORR activity of Co/NCS catalysts. The E_1/2_ of the Co/NCS-1, Co/NCS-2, Co/NCS-3, and Co/NCS-4 catalysts are found to be 0.52, 0.57, 0.63 and 0.59 V vs. RHE, respectively. It is clearly evident that incorporation of Co could enhance the ORR activity by creating Co-N-C/Co-S_x_, Co-N_x_ active sites as ascertained from the XPS analysis. Among the four Co/NCS catalysts, the Co/NCS-3 catalyst showed the optimum ORR activity with the E_1/2_ of 0.63 V vs. RHE. Therefore, quantitatively, a gain of 223 mV overpotential have been enhanced by Co metal active centers over NCS catalyst. The ORR activity of Co/NCS catalyst is compared with the commercial Pt/C (10 wt.% catalyst as shown in Figure 6b. The Pt/C catalyst exhibits the half wave potential of 0.72 V vs. RHE. Still, Pt/C catalyst show higher activity (~ catalyst loading of 110 µg cm^−2^) than the Co/NCS catalyst due to the fact that Pt is a noble metal with excellent ORR kinetics. The Co/NCS catalyst E_1/2_ is 90 mV lower than the Pt/C. Though, the gained ORR activity of Co/NCS is still encouraging due to the fact that Co based catalysts are cheaper than the noble metal catalyst.

**Figure 7:**
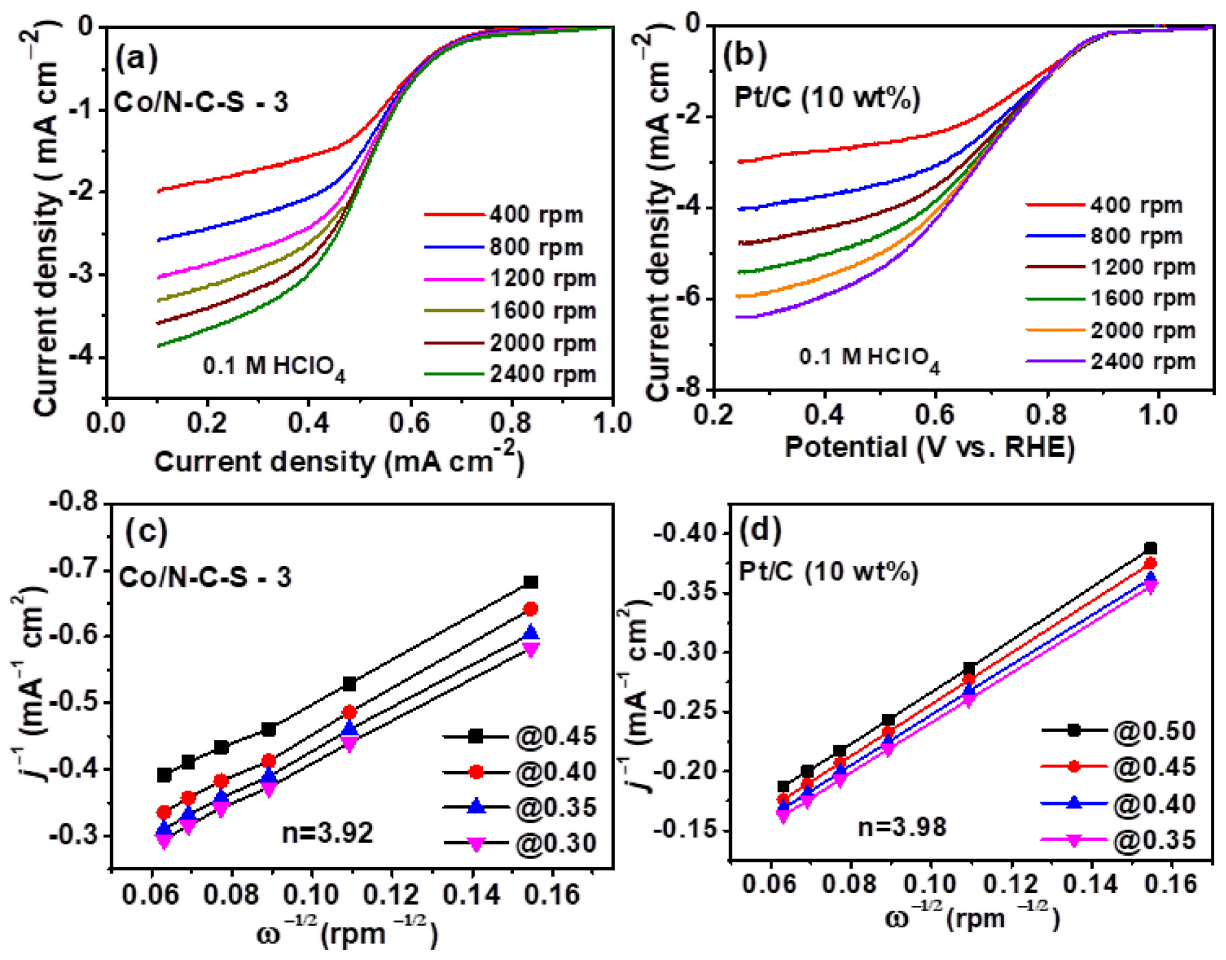
LSV curves of (**a**) Co/NSC-3 (**b**) Pt/C (10 wt.%) recorded at different rotation speeds and their respective K-L plots (**c**,**d**), respectively.

In order to gain knowledge on the ORR pathway, the LSV of Pt/C and Co/NCS catalysts are recorded at different rotations (Figure 7a,b) and K-L plots (Figure 7c,d) are derived. It is well known that ORR precedes either a direct 4 electron reduction process or by a 2 + 2 electron reduction process. The later pathway produces the undesired H_2_O_2_. The number of electrons involved in the ORR process is calculated from the slope of K-L plots, i.e., plots of inverse of square root of rotation speed vs. inverse of the current density. With no discernible change in the onset potential, it is shown that both catalysts exhibit increasing ORR limiting currents with regard to an increase in rpm, indicating that the ORR reaction is an O_2_ diffusion-controlled reaction (Figure 7a,b). Additional linearity in K-L graphs shows that electron transport kinetics at various potentials are virtually identical (Figure 7c,d). The number of electrons (n) were calculated to be 3.98 and 3.92 for Pt/C and Co/NCS catalysts, respectively. The obtained ‘n’ close to 4, clearly suggests that the catalyst perform ORR via a dominant, direct 4 electron pathway albeit a minor proportion of 2 + 2 electron reduction is still possible.

**Figure 8:**
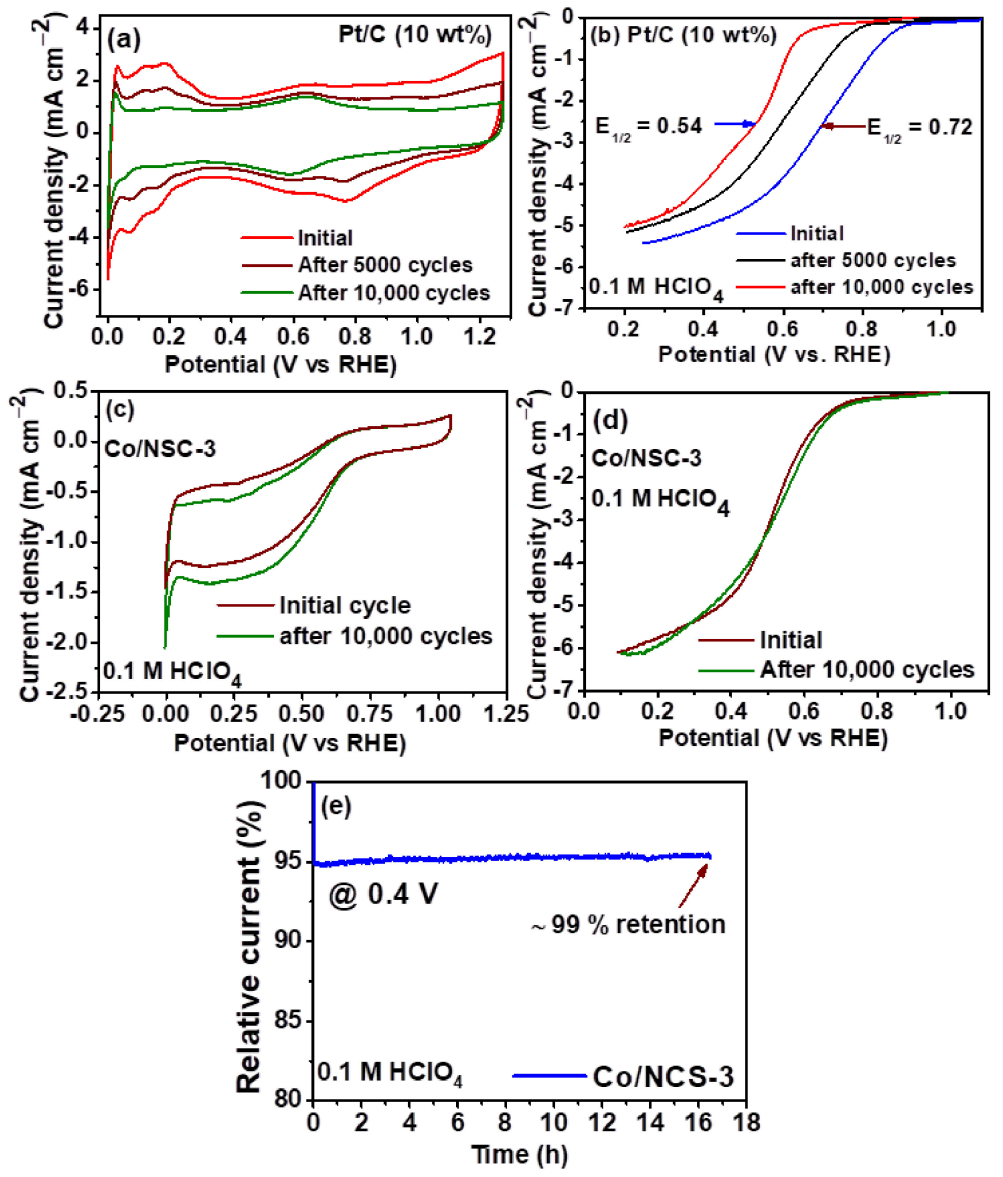
Cyclic voltammograms of (**a**) Pt/C (10 wt.%) (**c**) Co/NSC-3 recorded before and after the stability test. LSV curves recorded after 5000 and/or 100,000 potential cycles of cyclic voltammograms for (**b**) Pt/C (10 wt.%) (**d**) Co/NSC-3. (**e**) Chronoamperometric responses of Co/NSC-3 in O_2_ saturated electrolyte. Electrolyte: 0.1 M HClO_4_, scan rate: 50 mV/sec (CV) 10 mV/sec (LSV).

Further, the stability of Co/NCS-3 catalyst is then evaluated by potential cycling test by cyclic voltammetry technique. The catalyst stability is evaluated by taking LSV curve after the potential cycling test (Figure 8a–d). During the stability test analysis, the Pt/C catalyst showed a drastic decrease in the hydrogen adsorption/desorption region as can be seen from Figure 8a. This decrease in the currents of hydrogen adsorption/desorption region represents loss of electrochemical active surface area (ECSA) due to coalescence, Pt nanoparticles agglomeration induced by carbon corrosion [54]. This decrease in the ECSA causes less Pt surface exposure for the ORR catalysts therefore reducing the ORR activity. This was clearly established from the LSV curves recorded after 5000 and 10,000 potentials cycles as shown in Figure 8b. The half-wave potential of the Pt/C decreases by 180 mV after 10,000 potential cycles. Under similar experimental conditions, the Co/NSC-3 catalyst exhibited admirable stability. Figure 8c,d shows the cyclic voltammograms and LSV voltammograms of the Co/NCS-3 catalyst before and after the potential cycles, respectively. It is clearly seen the E_1/2_ of the Co/NCS-3 catalyst is hardly changed after 10,000 potential cycles, indicating that Co/NCS-3 catalyst have admirable stability. This significant stability of Co/NCS-3 is due to the interaction of Co nanoparticles with the N and S doped carbon support. To verify the stability of CO/NSC-3, we have analyzed the morphology of the Co/NSC-3 catalyst after the stability test, as shown in Figure S3. It is seen that Co nanoparticles seem disordered and along with slightly increased Co particle size after the stability test, due to potential cycling. However, the Co nanoparticles are still seen after the stability test, indicating that Co/NSC-3 catalyst is durable and stable against potential cycling. It is well established that carbon that has been doped with N and S has a high resistance to carbon corrosion during potential cycling. In addition, N-doped carbons offers anchoring effect to the metal nanoparticles, that alleviates the metal leaching in the acidic solution. Further, the stability of the Co/NCS catalyst is also evaluated by the constant voltage @ 0.4 V vs. RHE in 0.1 M HClO_4_ acidic solution as shown in Figure 8e. After a 17 h of the chronoamperometric measurement, the Co/NCS catalyst maintain ~99% of its initial current. This explains the excellent stability of the Co/NCS catalyst both under potential cycling test and chronoamperometric test.

**Figure 9:**
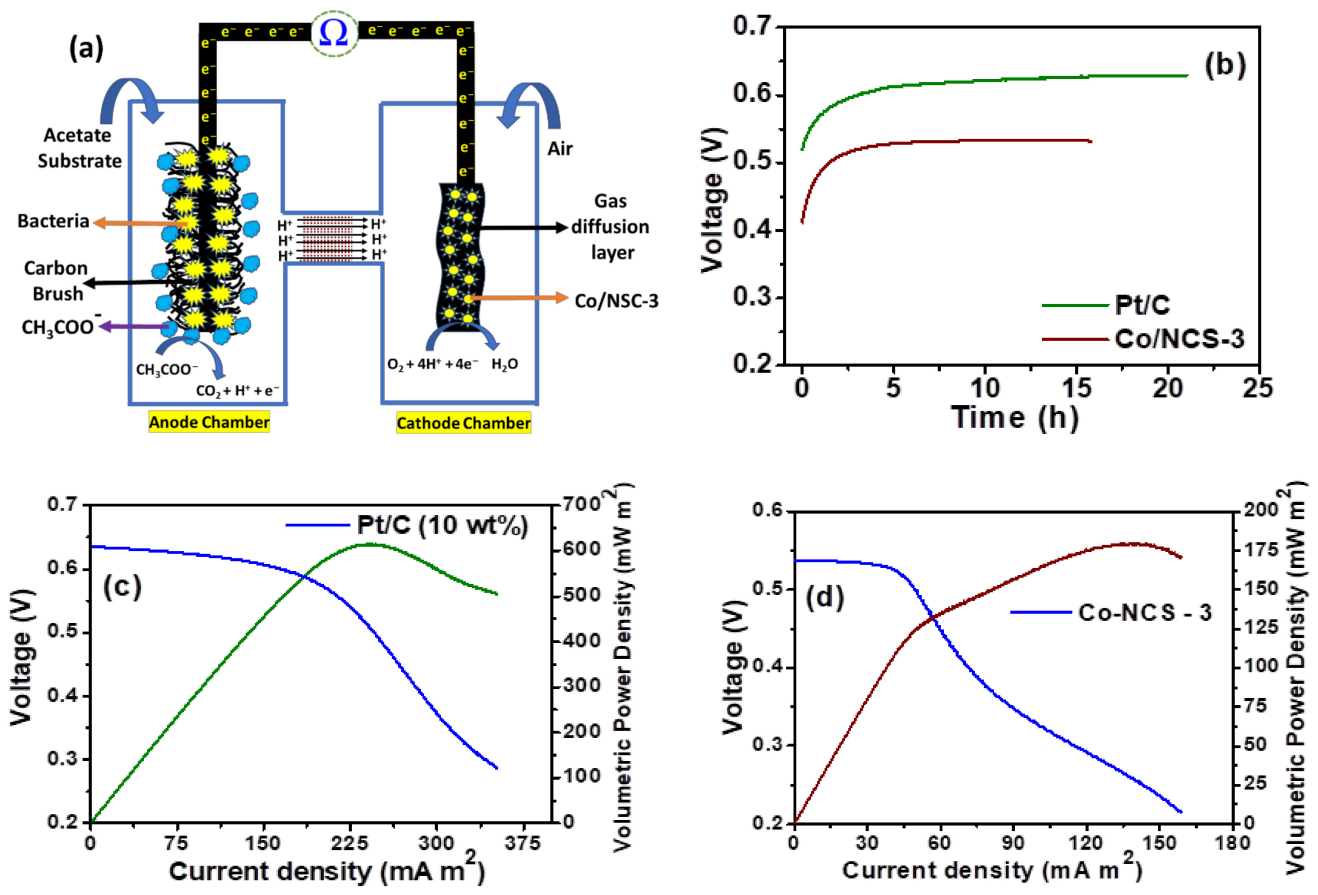
(**a**) Schematic representation of dual chamber microbial fuel cells (**b**) recorded OCV values of Co/NSC-3 and Pt/C (10 wt.%). The i-v characteristics of (**c**) Pt/C (10 wt.%) (**d**) Co-NSC-3 catalysts in dual chamber microbial fuel cells. Anolyte: 20 mM sodium acetate + pH 7 buffer, catholyte: Air saturated pH 7 buffer.

After ascertaining ORR activity of Co/NCS-3 catalyst, it is applied as cathode and its deliverable power density performance was evaluated in a dual chamber MFC set up as shown in in Figure 9a. The Co/NCS-3 catalyst is coated on the commercial gas diffusion layer at the catalyst loading of 2 mg cm^−2^. At first the open circuit voltage (OCV) was recorded by introducing Co/NSC-3 coated GDL into the cathode chamber of the already activated dual chamber MFC. It can be seen that the starting voltage of 412 mV is seen as soon as the catalyst is introduced into the cathode chamber. The potential slowly rose to a maximum 533 mV after 5 h of time and the voltage stayed relatively constant for about 15 h, indicating that the maximum voltage was achieved. Under similar conditions, Pt/C coated gas diffusion layer is also used as a cathode catalyst, which showed a starting voltage of 523 mV and the potential rose to maximum of 630 mV at the end time of 20 h (Figure 9b).

After achieving a stabilized OCV, the i–v curves are recorded to obtain the achievable power density of Pt/C and Co-NSC-3 catalysts as shown in the (Figure 9b–d). A volumetric power density of about 180 mW m^2^ was achieved by using Co-NSC-3 catalyst as cathode, catalyst, whereas Pt/C catalyst delivered a volumetric power density of ~615 mW m^2^. The difference in the obtained power density was reflected from the RDE studies, where Pt/C catalyst still showed better performance than the Co/NCS-3 catalyst. However, the power density of Co/NSC-3 catalyst can be further increased with the increased catalyst loading to match the performance of Pt/C. Increasing the catalyst loading of Co/NSC-3 is still justified, because of the lower cost of cobalt than the Pt/C.

## 4. Conclusions

Developing cheap, cost-effective catalysts is essential for the successful commercialization of the MFC. We have successfully synthesized the earth’s abundant Co based catalyst by a solid-state synthesis process by utilizing Co nitrate, Zinc nitrate and 2-aminothiazole. Pyrolysis of the (ZnxCo(C_3_H_4_N_2_S)) resulted in Co nanoparticles supported on the N and S doped carbons (Co/NSC). The TEM analysis clearly indicates that Co nanoparticles are evenly distributed on the N and S doped carbon support. The Co/NSC catalyst also showed the porous nature of the N and S doped carbons resulted from the evaporation of metallic Zn during the pyrolysis process. The XPS analysis shows the presence of catalytically actives phases of Co in the form of CoN_x_/CoS_x_/metallic Co species. RDE results reveals that Co/NSC-3 catalyst showed optimized ORR activity. The Co/NSC catalyst also showed excellent stability over 10,000 potential cycles and in chronoamperometric studies. In the DCMFC, the Co/NSC catalyst delivered a volumetric power density of ~180 mW m^2^.

## Data Availability

Not applicable.

## Supplementary Materials

The following supporting information can be downloaded at: https://www.mdpi.com/article/10.3390/nano12244369/s1, Details of the commercial GDL used as backing layers for both anodes and cathodes. Figure S1: Particle size distribution of Co nanoparticle in Co-NSC-3 catalysts derived from the TEM images.; Figure S2: (a) XPS survey spectrum for the Co-NSC-3 catalyst (b–d), survey scan of Zn 3p, 3s and 2p spectra, respectively. (e) Zn 2p high resolution scan. Figure S2.T: Elemental composition of Co-NSC-3 catalyst obtained from XPS analysis; Figure S3. TEM images of Co/NSC-3 catalysts after stability test.
