# Supplementary material for: Solid-State Synthesis of Cobalt/NCS Electrocatalyst for Oxygen Reduction Reaction in Dual Chamber Microbial Fuel Cells
Source: Nanomaterials (Basel). 2022 Dec 7;12(24):4369. doi: 10.3390/nano12244369 (PMC9788303; doi:10.3390/nano12244369)
Supplement: Supplementary file 1 [file nanomaterials-12-04369-s001.zip › nanomaterials-2051654-supplementary.pdf]

Supplementary Materials

Details of the commercial GDL used as backing layers for both anodes and cathodes:

- SIGRACET® GDL BC 35 as gas diffusion layers (GDL).
- The specifications are as follows
- Type: solid (paper)
- Thickness: 325 μm
- Basic weight: 110 g/m^2
- Air permeability: 1.5cm3/(cm2*s)
- Through plane resistance: <15 mΩ cm^2
- Teflon (PTFE %) content: 30

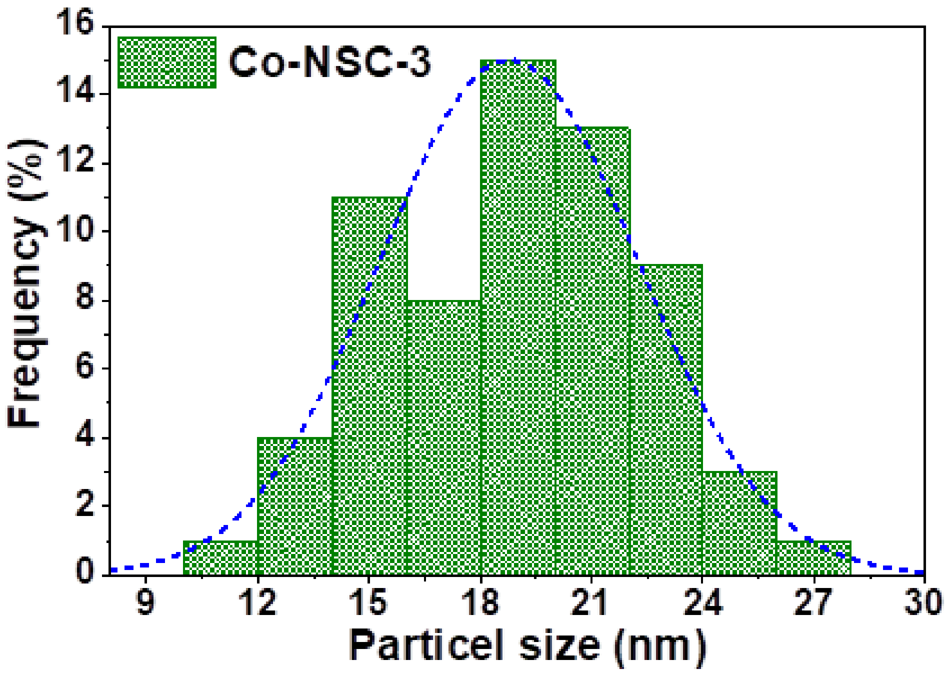

Figure S1. Particle size distribution of Co nanoparticle in Co-NSC-3 catalysts derived from the TEM images.

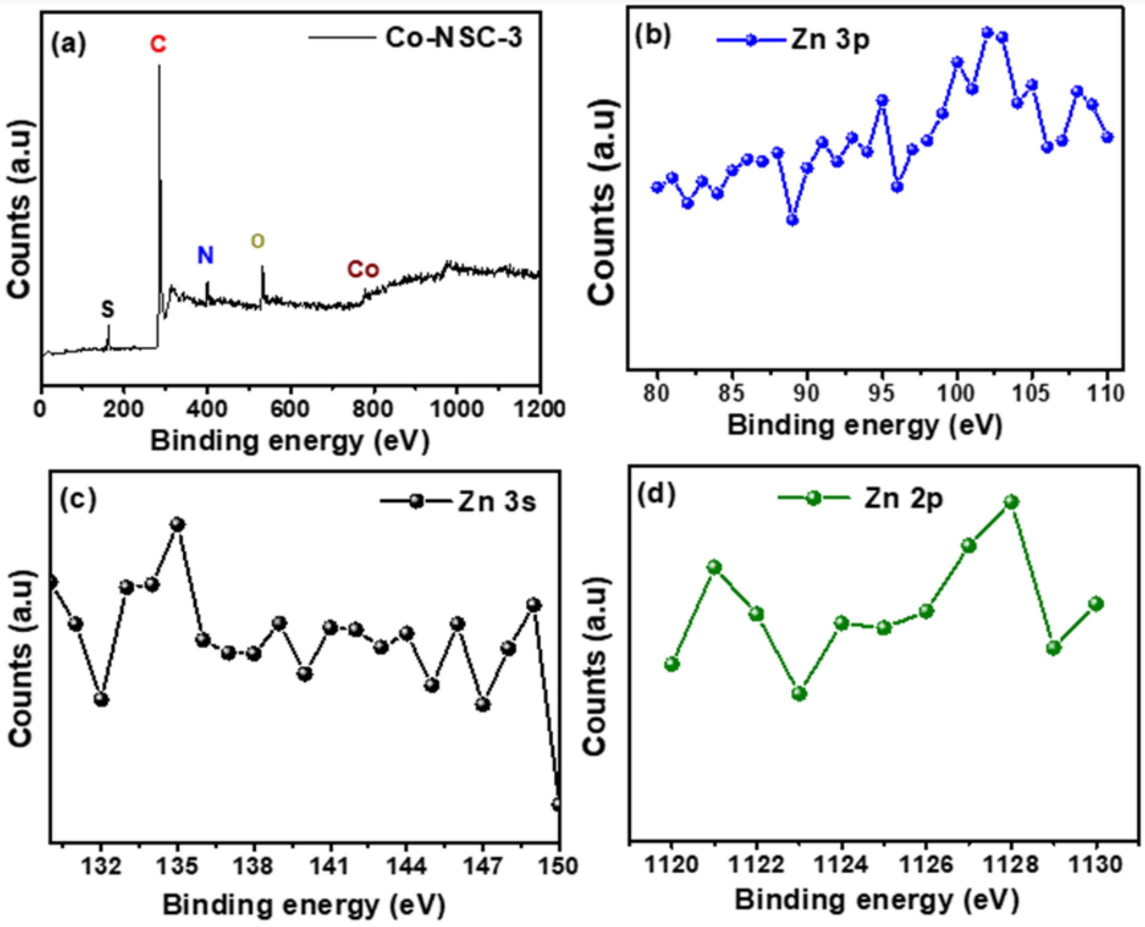

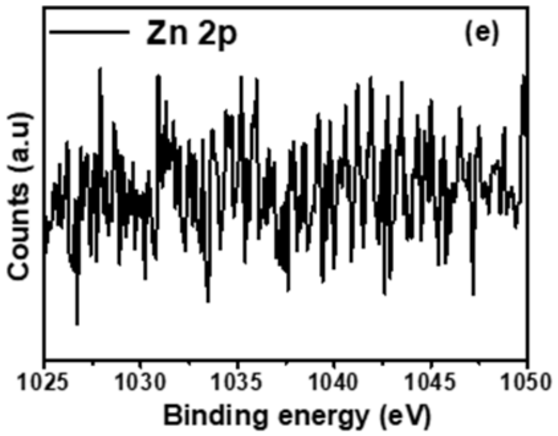

Figure. S2.T.

From XPS						
Element	C	O	N	S	Co	Zn
At%	51.02	38.23	5.04	2.57	3.10	0.04

Figure S2. (**a**) XPS survey spectrum for the Co-NSC-3 catalyst (**b–d**), survey scan of Zn 3p, 3s and 2p spectra, respectively. (**e**) Zn 2p high resolution scan. Figure. S2.T.

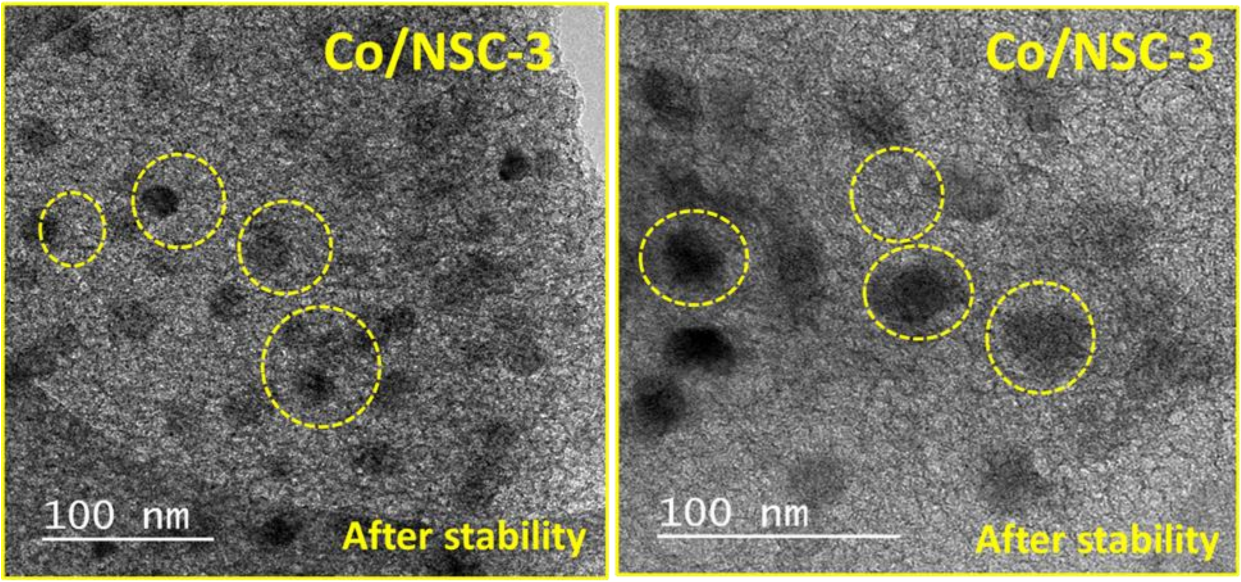

Figure S3. TEM images of Co/NSC-3 catalysts after stability test.
